# Influence of Genetic Risk Factors on Coronary Heart Disease Occurrence in Afro-Caribbeans

**DOI:** 10.1016/j.cjca.2016.01.004

**Published:** 2016-08

**Authors:** Laurent Larifla, Katherine E. Beaney, Lydia Foucan, Jacqueline Bangou, Carl T. Michel, Jean Martino, Fritz-Line Velayoudom-Cephise, Jackie A. Cooper, Steve E. Humphries

**Affiliations:** a Research Group Clinical Epidemiology and Medicine, ECM/L.A.M.I.A EA 4540, University Hospital of Pointe-à-Pitre, University of Antilles, Guadeloupe, France; b Centre for Cardiovascular Genetics, British Heart Foundation Laboratories, Institute of Cardiovascular Sciences, University College London, London, United Kingdom; c Department of Cardiology, University Hospital of Pointe-à-Pitre, Guadeloupe, France

## Abstract

**Background:**

Despite excessive rates of cardiovascular risk factors such as hypertension, diabetes, and obesity, Afro-Caribbeans have lower mortality rates from coronary heart disease (CHD) than do whites. This study evaluated the association of genetic risk markers previously identified in whites and CHD in Afro-Caribbeans.

**Methods:**

We studied 537 Afro-Caribbean individuals (178 CHD cases and 359 controls) who were genotyped for 19 CHD-related single-nucleotide polymorphisms (SNPs). A genetic risk score (GRS) incorporating the 19 SNPs was calculated. These participants were compared with 1360 white individuals from the Second Northwick Park Heart Study.

**Results:**

In Afro-Caribbeans, patients with CHD had higher rates of hypertension (78.7% vs 30.1%), hypercholesterolemia (52.8% vs 15.0%), and diabetes (53.9% vs 14.8%) and were more often men (64.0% vs 43.7%) and smokers (27.5% vs 13.4%) compared with non-CHD controls (all *P* < 0.001). The GRS was higher in Afro-Caribbeans with CHD than in those without CHD (13.90 vs 13.17; *P* < 0.001) and was significantly associated with CHD after adjustment for cardiovascular risk factors, with an odds ratio of 1.40 (95% confidence interval, 1.09-1.80) per standard deviation change. There were significant differences in allelic distributions between the 2 ethnic groups for 14 of the 19 SNPs. The GRS was substantially lower in Afro-Caribbean controls compared with white controls (13.17 vs 16.59; *P* < 0.001).

**Conclusions:**

This study demonstrates that a multilocus GRS composed of 19 SNPs associated with CHD in whites is a strong predictor of the disease in Afro-Caribbeans. The differences in CHD occurrence between Afro-Caribbeans and whites might be a result of significant discrepancies in common gene variant distribution.

## Résumé

**Introduction:**

En dépit des taux excessifs des facteurs de risque cardiovasculaire comme l'hypertension, le diabète et l'obésité, les Afro-Caribéens ont des taux de mortalité liée aux maladies coronariennes (MC) plus faibles que les Blancs. La présente étude évaluait l'association entre les marqueurs de risque génétique précédemment déterminés chez les Blancs et les MC chez les Afro-Caribéens.

**Méthodes:**

Nous avons étudié 537 personnes afro-caribéennes (178 cas de MC et 359 témoins) chez qui le génotypage de 19 polymorphismes de nucléotide simple (SNP : *single-nucleotide polymorphism*) liés à la MC avait été effectué. Nous avons calculé un score de risque génétique (SRG) qui incorpore les 19 SNP. Nous avons comparé ces participants aux 1360 individus blancs de la deuxième étude Northwick Park Heart Study.

**Résultats:**

Chez les Afro-Caribéens, les patients atteints de MC avaient des taux plus élevés d'hypertension (78,7 % *vs* 30,1 %), d'hypercholestérolémie (52,8 % *vs* 15,0 %) et de diabète (53,9 % *vs* 14,8 %), et étaient plus souvent des hommes (64,0 % *vs* 43,7 %) et des fumeurs (27,5 % *vs* 13,4 %) que les témoins qui n’étaient pas atteints de MC (tous *P* < 0,001). Le SRG était plus élevé chez les Afro-Caribéens atteints de MC que chez ceux qui n’étaient pas atteints de MC (13,90 *vs* 13,17; *P* < 0,001) et significativement associé aux MC après l'ajustement des facteurs de risque cardiovasculaire, et montrait un ratio d'incidence approché de 1,40 (intervalle de confiance à 95 %, 1,09-1,80) par changement d’écart type. Les distributions des fréquences alléliques de 14 des 19 SNP montraient des différences significatives entre les 2 groupes ethniques. Le SRG était substantiellement plus bas chez les témoins afro-caribéens que chez les témoins blancs (13,17 *vs* 16,59; *P* < 0,001).

**Conclusions:**

Cette étude démontre que le SRG multilocus composé de 19 SNP qui est associé aux MC chez les Blancs est un fort prédicteur de la maladie chez les Afro-Caribéens. Les différences dans la survenue des MC entre les Afro-Caribéens et les Blancs pourraient résulter des divergences importantes dans la distribution des variants génétiques fréquents.

Coronary heart disease (CHD) is the leading cause of death in most industrialized countries, with a growing epidemic in low and middle income countries.^1^ The development of the disease depends on a complex interaction between environmental and genetic factors. Modifiable risk factors such as hypertension, dyslipidemia, diabetes, obesity, and smoking can predict 80%-90% of the risk of CHD in most populations around the world.^2^ However, wide differences in CHD incidence and mortality rates between populations or ethnic groups are not fully explained by their respective distribution of traditional cardiovascular risk factors (TCRFs). Indeed, some populations may have a lower burden of CHD than others despite similar rates of TCRFs.^3^, ^4^

The epidemiology of CHD in Afro-Caribbean individuals is a striking example of the disproportionate relation between CHD occurrence and TCRF distribution at the population level. In the French Caribbean island of Guadeloupe, approximately 80%-85% of the population is of Afro-Caribbean origin. Although there are few smokers (15%), there is a high prevalence of hypertension (30%), diabetes (10%), and obesity (23%).^5^, ^6^ However, observational data indicate lower standardized mortality rates from CHD in both men (–50%) and women (–40%) compared with mainland France, which has one of the lowest incidences of CHD in Europe.^7^, ^8^, ^9^ Similar differences between white and Afro-Caribbean communities have also been reported in the United Kingdom and remain unexplained.^9^, ^10^ The study of such ethnic differences is important not only for the concerned populations but also for all populations, because it can provide key information for the management of CHD and identify new targets for treatment and preventive strategies.

The lack of a strong correlation between CHD incidence and TCRFs suggests that genetic variation could lie behind the interpopulation variability in coronary mortality. In the past few years, several susceptibility loci for CHD have been identified; mostly in white populations, however, no data are available in Afro-Caribbean populations. Genetic risk scores (GRSs) could be a valuable tool to assess and compare the genetic susceptibility for complex diseases in different ethnic groups, because they summarize the potential genetic influences across multiple loci into a single quantitative parameter.^11^ In this study, we aimed to describe in Afro-Caribbeans with and without CHD, the allelic distribution of 19 CHD-related single nucleotide polymorphisms (SNPs) that have been reported in white studies. We also tested the hypothesis that the differences in genetic susceptibility for CHD assessed by a GRS could contribute to the disparity in CHD occurrence between whites and Afro-Caribbeans.

## Methods

### Study populations, CHD status, and TCRF definition

The Afro-Caribbean participants were recruited from the island of Guadeloupe, a French Caribbean region with a wide availability of health services and easy access to medical care regardless of income. All participants were Afro-Caribbeans. The ethnic origin was determined when the patient defined him or herself and 2 first-degree relatives as Afro-Caribbean. Cases were selected from the Department of Cardiology at the University Hospital of Pointe-à-Pitre. Patients were eligible if they had a documented history of previous acute myocardial infarction according to the World Health Organization criteria or coronary bypass surgery (or both) or coronary angioplasty. Control participants were selected from a public health centre on the same island and who had no suspicion or history of cardiovascular disease. TCRFs were determined using the same definition in cases and controls. Participants were considered as having hypertension if they had a history of hypertension or were prescribed antihypertensive therapy. Similarly, they were considered as having diabetes or hypercholesterolemia if they had a history of diabetes or hypercholesterolemia or were prescribed hypoglycemic agents (including insulin) or lipid-lowering therapy, respectively. Participants were considered to be current smokers if they were regularly smoking more than 1 cigarette per day at the time of inclusion. Body mass index (BMI) was calculated as weight/height^2^ (kg/m^2^), and obesity was determined by a BMI ≥ 30 kg/m^2^. All participants gave their written informed consent to participate in the study, which received the approval of the inter-regional ethics committee (Sud-Ouest/Outre-Mer III, France).

The white sample used was from the Second **N**orthwick **P**ark **H**eart **S**tudy (NPHSII) and has already been described.^12^ Briefly, 3052 middle-aged men (50-64 years of age) with no previous history of cardiovascular disease were recruited from 9 general practices in the United Kingdom. The study started in 1989, and incident cases of acute or silent myocardial infarction and coronary surgery were recorded during a median of 13.5 years of follow-up. All participants gave written informed consent, and the study was approved by the National Health Service Health Research Authority Research Ethics Committee London–Central.

### Genotyping and genetic scores calculation

Genotyping was performed using published TaqMan (Applied Biosciences/Life Technologies, Grand Island, NY), and KASPar (KBioscience, Hertfordshire, UK) technologies. We selected 19 SNPs that were associated with CHD in whites in genome wide association studies (GWASs) or meta-analysis of candidate gene association studies.^13^, ^14^ A nonweighted GRS was calculated for each individual by summing the number of risk alleles at each locus (0 if risk allele was absent and 1 if heterozygote or 2 if homozygote for the risk allele). A weighted score was calculated by multiplying the number of risk alleles at each locus by the corresponding β coefficient from the CARDIoGRAMplusC4D meta-analysis data (or the CARDIoGRAM GWAS if this was not available). Data on coronary artery disease/myocardial infarction were contributed by CARDIoGRAMplusC4D investigators and downloaded from www.cardiogramplusc4d.org/. Regarding the nonweighted score, we assumed that all SNPs were acting in an additive manner. We previously reported that a weighted gene score including these SNPs was associated with CHD in the NPHSII cohort.^15^

We also calculated (using the same method) a 14-SNP GRS after exclusion of SNPs in loci at which the *P* value for association with CHD was < 0.01 in either the CARDIoGRAM GWAS or CARDIoGRAMplusC4D meta-analysis.^14^, ^16^ The SNPs included in the scores and their estimates are presented in Table 1.

### Statistical analysis

Only participants with complete data regarding TCRFs and genotype were included for the analysis. In the Afro-Caribbean sample, we randomly selected 2 controls for each case. A χ^2^ test was used to assess whether each SNP was in Hardy-Weinberg equilibrium. The GRSs were considered as continuous variables. We used logistic regression to assess the association of GRSs and TCRFs with CHD as a binary dependant variable. To evaluate the predictive value of GRS in Afro-Caribbeans, we used the receiver-operating characteristic curve analysis and calculated the area under the curve from logistic models adjusted by age and sex. IBM SPSS Statistics, version 21 (SPSS, Chicago, IL) was used for analysis. All tests were 2 sided and *P* values of < 0.05 were considered significant.

## Results

In the Afro-Caribbean sample, the analysis included a total of 178 cases and 359 controls. Table 2 shows the characteristics of the participants. Patients with CHD had higher rates of hypertension, diabetes, and hypercholesterolemia and were more often men and smokers than were controls. The prevalence of obesity and the mean BMI did not differ between cases and controls.

For the 19 genotyped SNPs, call rates ranged from 94%-98%. All SNPs were in Hardy-Weinberg equilibrium. The risk allele frequency of rs599839 (*CELSR2/PSRC1/SORT1*) and rs1746048 (*CXCL12*) was higher in cases than in controls (0.35 vs 0.25; *P* = 0.01 and 0.63 vs 0.53; *P* = 0.03, respectively). The difference was nearly significant for rs17465637 (*MIA3*): 0.31 in cases vs 0.24 in controls (*P* = 0.08). Full details are available in Supplemental Table S1.

### Differences in common CHD-related SNPs between Afro-Caribbeans and whites

Table 3 shows the risk allele frequencies of the 19 SNPs in white and Afro-Caribbean participants without CHD. For rs11591147, rs1801177, and rs3798220, comparisons were not meaningful because the minor allele frequencies were very low or null in both groups. For the remaining SNPs, there were significant differences in allelic distributions between the 2 ethnic groups, with the exception of rs7412 (*APOE*) and rs1042031 (*APOB*). Both the mean nonweighted GRS19 and GRS14 were lower in Afro-Caribbean controls than in white controls: 13.17 vs 16.59 (*P* < 10^−100^) and 9.69 vs 12.76 (*P* < 10^−100^), respectively (Table 3). Histograms representing the distribution of the nonweighted GRS in white and Afro-Caribbean controls are shown in Supplemental Figure S1.

### Association between GRS and CHD in Afro-Caribbeans

The weighted and the nonweighted GRS19 were significantly higher in Afro-Caribbeans with CHD than in those without CHD: 13.90 (± 2.07) vs 13.17(± 2.10); *P* = 1.6 × 10^−4^ and 1.74 (± 0.24) vs 1.68 (± 0.20); *P* = 0.01, respectively. Results were similar with the weighted and the nonweighted GRS14 (Supplemental Table S2).

**Figure 1:**
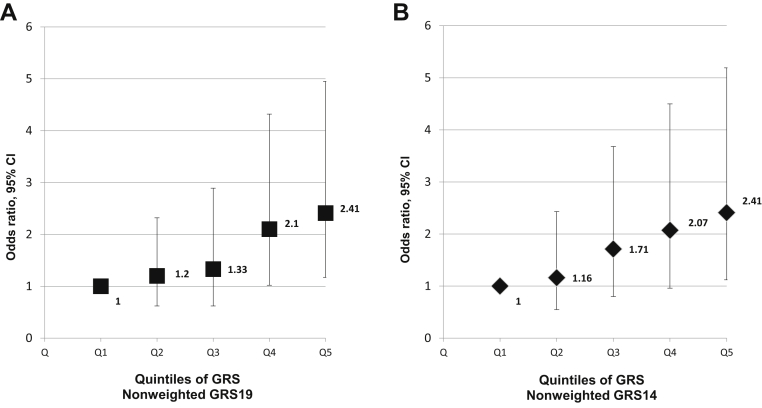
Association between genetic risk score (GRS)19 (**A**) and GRS14 (**B**) divided into quintiles and coronary heart disease (CHD) in Afro-Caribbeans. Each quintile from the first (Q1) to the fifth (Q5) are shown along the x axis. Odds ratios with the **error bars** representing the 95% confidence interval are plotted on the y axis and were calculated from logistic regression models incorporating nonweighted GRS, age, sex, hypertension, diabetes, hypercholesterolemia, and smoking. First quintile: reference group. For GRS19: second quintile, *P* = 0.60; third quintile, *P* = 0.60; fourth quintile, *P* = 0.04; fifth quintile, *P* = 0.02. For GRS14: second quintile, *P* = 0.70; third quintile, *P* = 0.17; fourth quintile, *P* = 0.06; fifth quintile, *P* = 0.02.

After adjustment for age, sex, diabetes, hypertension, hypercholesterolemia, and smoking, the association between GRS and CHD was not significant for the weighted GRS19, nearly significant for the weighted GRS14, and highly significant for both nonweighted GRSs, with odds ratios (ORs) of 1.40 (95% confidence interval [CI], 1.09-1.80; *P* = 0.008) for GRS19 and 1.43 (95% CI, 1.12-1.83; *P* = 0.004) for GRS14 (Tables 4 and 5). In the model incorporating the TCRFs, analysis between subsequent quintiles of the nonweighted GRS19 showed a stepwise increase in the risk of CHD from the first to fifth quintiles. Participants in the top quintile (11.7% of the controls) had an OR of 2.41 (95% CI, 1.17-4.95; *P* = 0.02) compared with those in the bottom quintile (38.2% of the controls). Results were similar when using the nonweighted GRS14 (Fig. 1).

**Figure 2:**
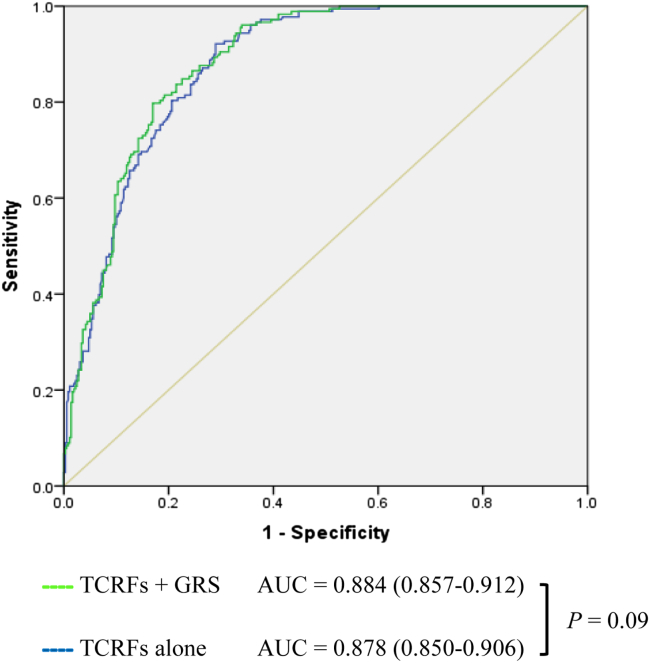
Incremental contribution of genetic risk score (GRS) to coronary heart disease (CHD) discrimination in Afro-Caribbeans (n = 537). Predictions are based on logistic regression models incorporating traditional cardiovascular risk factors (TCRFs) (age, sex, hypercholesterolemia, diabetes, hypertension, and smoking) alone or with the addition of the nonweighted 14 single nucleotide polymorphism (SNP) GRSs. The area under the curve (AUC) measures the ability of the model to discriminate between individuals with CHD and those without CHD.

Figure 2 shows the receiver operating characteristic curve area under the curve (AUC) for the logistic regression models incorporating TCRFs alone or with addition of the nonweighted GRS14. All models were adjusted for age. The addition of the GRS appeared to slightly increase the AUC from the model incorporating only TCRFs, but the difference was not significant: *P* = 0.09 for GRS14 and *P* = 0.2 for GRS19 (Supplemental Fig. S2).

### Association between GRS and TCRFs in Afro-Caribbeans

We compared the mean GRS19 in the CHD and non-CHD participants separately according to the presence or absence of each TCRF. In the non-CHD group, both the weighted and the nonweighted GRS19 did not differ in patients with diabetes and those without diabetes, and results were similar when hypertension, hypercholesterolemia, or smoking was considered (Supplemental Table S3). In the CHD group, the weighted and nonweighted GRS19 were not associated with hypertension, smoking, or diabetes, but both were significantly higher in patients with hypercholesterolemia compared with those without hypercholesterolemia (Supplemental Table S4).

### Assessment of the risk of CHD in Afro-Caribbeans and whites based on the GRS

To compare the strength of the association between the GRSs and CHD in Afro-Caribbeans and whites, we calculated the OR of weighted and nonweighted GRS19 and GRS14 in models incorporating TCRFs in both ethnic groups. For this analysis, the Afro-Caribbean women (n = 266) were excluded because the NPHSII cohort included only men. As shown in Table 6, the ORs of the GRSs were similar in Afro-Caribbeans and whites.

The mean nonweighted GRS19 and nonweighted GRS14 were significantly lower in Afro-Caribbean men than in whites: 12.88 (± 2.00) vs 16.59 (± 2.20) and 9.53 (± 1.66) vs 12.76 (± 1.69) (*P* < 10^−100^for all). Based on these observed differences in GRS and on the predictions from the logistic models incorporating risk factors (age, hypertension, diabetes, hypercholesterolemia, and smoking), the expected risk for CHD would be reduced by 54% or 44% in Afro-Caribbeans compared with whites depending on the use of the GRS19 or the GRS14, respectively.

## Discussion

In this study, we investigated the association between 19 selected CHD genetic risk markers, TCRFs, and CHD in Afro-Caribbeans. We also assessed if the differences in risk allele frequency between Afro-Caribbeans and whites are a potential explanation for the lower occurrence of CHD reported in Afro-Caribbeans.

We found that a GRS representing the cumulative effect of 19 SNPs was significantly associated with CHD in Afro-Caribbeans. Moreover, the contribution of the GRS to CHD prediction was high and, based on the logistic regression analysis, provided complementary information beyond TCRFs. There were marked differences in the allelic distributions of the 19 SNPs between whites and Afro-Caribbeans, and both the weighted and nonweighted GRSs were substantially and significantly lower in Afro-Caribbeans than in whites, suggesting that the lower CHD risk seen in Afro-Caribbeans may result in part from a lower prevalence of these CHD risk alleles.

### Association of TCRFs with CHD in Afro-Caribbeans

Observational studies have documented a lower mortality rate from CHD in Afro-Caribbeans living in the Caribbean as well as in those living in Europe.^7^, ^10^, ^20^ However, few studies have investigated the association between TCRFs and CHD in this population or examined the reason for the low mortality from CHD in Afro-Caribbeans despite a high prevalence of risk factors such as diabetes, hypertension, and obesity seen in the general population.^9^, ^21^, ^22^ Some studies have suggested a more favourable lipid profile, even in the context of insulin resistance, compared with whites and also a lower impact of diabetes on CHD in Afro-Caribbeans than in whites or South Asians.^21^, ^23^ Conversely, in the INTERHEART case-control study involving 52 countries around the world, the ORs for the association of the major TCRFs with CHD did not vary appreciably among ethnic groups.^2^ However, no Afro-Caribbean sample was included in this study. In our study, the prevalence of TCRFs in the control group were in line with those previously reported in the general population, indicating that our sample was representative of this population.^5^, ^6^ In the Afro-Caribbean cases, the presence of all major risk factors was 2-3.5 times higher than in the controls except for obesity. This finding does not support the hypothesis that a lower impact of TCRFs could fully explain the low rate of CHD in Afro-Caribbeans and reinforces the need for an alternative or complementary explanation.

### Gene variant for CHD in Afro-Caribbeans

To the best of our knowledge, this is the first study on the allelic distribution of these CHD-related SNPs in Afro-Caribbeans. For most SNPs, the allelic distribution was markedly different than in whites and similar to or approximately midway between that of the HapMap samples from the Yoruba of Nigeria and that of African Americans (Supplemental Table S5). Only 2 SNPs, rs599839 (*CELSR2*/*PSRC1*/*SORT1*) and rs1746048 (*CXCL12*), were statistically associated with CHD. However, it is highly likely that a lack of power because of our sample size would mask other potential associations. The direction of the association was the same as in previous GWASs performed in whites. Although it is likely that most of the reported GWAS-lead SNPs are not the causal variant but are only markers in linkage disequilibrium (LD) with the functional variant, 11 of the variants included in this SNP panel directly affect the function or level of the cognate protein (eg, the common *APOE* variant). Nonetheless, given the likely differences in LD structure between the 2 ethnic groups, it is possible that some of the selected SNPs associated with CHD in whites would be a poor marker (because of low LD with the causal variation) in Afro-Caribbeans.

### Association of the GRSs with CHD in Afro-Caribbeans

The weighted and nonweighted GRSs were significantly higher in Afro-Caribbean participants with CHD than in controls, even after adjustment for TCRFs. The nonweighted scores (GRS19 and GRS14) were strong predictors of CHD, with a 40% increase in CHD risk per standard deviation. Participants in the highest quintile of GRS had a nearly 2.5-fold higher risk of CHD compared with those in the lowest quintile. The weighted GRSs calculated on the basis of the risk estimates reported in Europeans appear to be less predictive of CHD than are the nonweighted GRSs. This is likely a consequence of differing LD patterns, in which some of the lead SNPs are in much weaker LD with the causal SNP in Afro-Caribbeans compared with whites. This will vary from SNP to SNP depending on the LD pattern at each risk locus. Weighting gene scores using β effects is used to reflect the relative contribution each locus has to risk (as opposed to treating them as having the same effect); however, if some SNPs are much better proxies than others, this will also be reflected and can result in a poorly fitting model.

### Usefulness of the GRS to assess interpopulation variation in CHD occurrence

Although being highly associated with CHD in Afro-Caribbeans, the GRSs were markedly lower in Afro-Caribbeans compared with whites. These 2 findings support our hypothesis that the differences in genetic susceptibility between the 2 populations could be an important contributor to the lower mortality from CHD in Afro-Caribbeans. It is assumed that 40%-60% of the risk of CHD is heritable,^24^ and most modifiable TCRFs (such as hypertension, dyslipidemia, diabetes, or obesity) are highly heritable, making a genetic approach an attractive way to assess the occurrence of this complex disease. However, the SNPs identified by GWASs confer individually a modest increase in relative risk (average OR of 1.15-1.20). Thus, the summation of each SNP effect in a single variable called the GRS has the ability to capture a larger part of the genetic susceptibility to the disease. Different GRS constructions have been used in many studies, but the GRS usually added only modestly to the disease prediction based on TCRF models.^11^, ^25^ In this study, we used the GRS to assess CHD risk within the Afro-Caribbean population but also through a comparison between 2 populations of different ancestry. Published works using this approach at an interpopulation level are scarce. Our data indicate that based on the observed differences in GRS between the 2 ethnic groups, the expected risk for CHD would be 44%-54% lower in Afro-Caribbeans than in whites, which is comparable to the 50% lower CHD mortality in these individuals reported in France and the United Kingdom.^7^, ^10^ Although we have demonstrated the potential utility of the nonweighted GRS within the Afro-Caribbean population, the data demonstrate that the published weighting from the white population reduces the predictive value of the GRS. This suggests that the weighting of SNPs for CHD risk cannot be directly transferred between different ethnic groups. To improve the clinical utility of the GRS, ethnic-specific weightings, obtained from large population-based cohorts, will be needed. Confirmatory studies are necessary to prove the utility of this tool to assess the disparity in CHD risk between populations, in particular when they are not fully explained by the distribution of TCRFs.

Interestingly, despite a genetic architecture similar to that of Afro-Caribbeans, African Americans have higher rates of CHD than Americans of European ancestry. In fact, in the first half of the 20th century, CHD mortality was lower in Afro-Americans than in European Americans but has declined less rapidly among African Americans than among European Americans and now are higher in African Americans than in other ethnic groups in the United States.^26^ The high prevalence of most CRFs in African Americans and the impact of a low socioeconomic status on heath and access to care are well-known confounding factors that could have overwhelmed the potential effects of a lower genetic burden in this population.

Our study has some limitations. The size of our Afro-Caribbean sample may have been insufficient to detect all potential associations between individual SNPs and CHD, and we cannot rule out that some SNPs with lower or even similar size effect as those highlighted in this study were not detectable in this sample. We assessed the difference in genetic burden between whites and Afro-Caribbeans using a constructed GRS and assuming that the risk for CHD was linked to the same set of genetic factors in both populations. Moreover, our 19-SNP selection represented only part of the list of the more robustly CHD-associated SNPs that comprise at least 46 loci to date,^14^ and no GWAS had been performed in Afro-Caribbeans. Taken together, these limitations make it possible that the gap in genetic burden between Afro-Caribbeans and whites could have been different with a wider GRS. However, to date, GWASs performed in African Americans, who have a genetic architecture similar to that of the Afro-Caribbean participants examined here, found no novel variant with reproducible association with CHD that had not been described in whites.^27^, ^28^

Hence, the high discrimination ability of the GRS in Afro-Caribbeans leads us to believe that a significant part of the CHD risk information has been captured with this approach in our study. We believe that the main limits of our study can be overcome in a future work testing a wider GRS in a larger multiethnic study designed to confirm the potential of this original approach. If confirmed, this approach could be of clinical and epidemiologic interest in assessing the risk for CHD in different populations in addition to the traditional risk factor assessment. In this study, the GRS was considered as a complementary tool rather than an alternative to traditional cardiovascular risk factor assessment. Because of the limited size of our sample and the lack of specific estimates for Afro-Caribbeans, we did not focus on the incremental value of the GRS in this study. Although incorporating a gene score test into a patient's clinical risk assessment using conventional risk factors would have cost implications, such costs would be minimal because the genotyping of the 19 SNPs used here can easily and cheaply be carried out using a saliva sample.

## Conclusions

This study demonstrates that a multilocus GRS composed of SNPs associated with CHD in whites is a strong predictor of the disease in Afro-Caribbeans. The genetic burden for CHD identified by this 19-SNPs GRS was significantly lower in Afro-Caribbeans than in whites and could be a major contributor to the disparity in CHD mortality between these 2 populations. Further work is needed to confirm these findings and to identify more extensively the genetic variants that contribute to CHD in Afro-Caribbeans.

## Disclosures

K.B. is supported by an MRC CASE award (1270920) with Randox Laboratories. L.L. is supported by Conseil regional de Guadeloupe (regional council of Guadeloupe). S.E.H. is a British Heart Foundation Professor, and he and J.C. are supported by the British Heart Foundation (RG008/08) and by the National Institute for Health Research University College London Hospitals Biomedical Research Centre. NPHSII was supported by the British Medical Research Council, and the US National Institutes of Health (grant number NHLBI 33014). The other authors have no conflicts of interest to disclose.

## Figures and Tables

**Table 1:** SNPs included in the genetic risk scores (GRS14 and GRS19) and their published estimates

SNP	Chromosome	Gene/locus	SNP location or amino acid change	Alleles	Published OR	Reference
Risk/reference
rs4341	17q23	*ACE*	Intergenic	G/C	1.01	Schunkert et al.^16^
rs662799∗	11q23	*APOA5*	Promoter Variant	G/A	1.1	CARDIoGRAMplusC4D Consortium et al.^14^
rs1042031	2p23-24	*APOB*	E4181K	A/G	1.01	CARDIoGRAMplusC4D Consortium et al.^14^
rs429358∗	19q13.2	*APOE*	C112R	C/T	1.25	Bennett et al.^17^
rs7412∗	19q13.2	*APOE*	C158R	C/T	1.06	Bennett et al.^17^
rs10757274∗	9p21	*CDKN2A/CDKN2B*	Intergenic	G/A	1.23	CARDIoGRAMplusC4D Consortium et al.^14^
rs599839∗	1p13.3	*CELSR2/PSRC1/SORT1*	Intergenic	A/G	1.11	CARDIoGRAMplusC4D Consortium et al.^14^
rs708272	16q21	*CETP*	Intronic	C/T	1.04	Schunkert et al.^16^
rs1746048∗	10q11.2	*CXCL12*	Intergenic	C/T	1.07	CARDIoGRAMplusC4D Consortium et al.^14^
rs7025486∗	9q33	*DAB2IP*	Intergenic	A/G	1.04	CARDIoGRAMplusC4D Consortium et al.^14^
rs10455872∗	6q26	*LPA*	Intergenic	G/A	1.32	Schunkert et al.^16^
rs3798220∗	6q26	*LPA*	I1891M	C/T	1.28	Schunkert et al.^16^
rs1801177∗	8p22	*LPL*	D36N	A/G	1.09	CARDIoGRAMplusC4D Consortium et al.^14^
rs328∗	8p22	*LPL*	S474X	C/G	1.1	CARDIoGRAMplusC4D Consortium et al.^14^
rs17465637∗	1q41	*MIA3*	Intergenic	C/A	1.14	Schunkert et al.^16^
rs9818870∗	3q23.3	*MRAS*	Intergenic	T/C	1.07	CARDIoGRAMplusC4D Consortium et al.^14^
rs1799983	7q35-36	*NOS3*	D298E	T/G	1.0	CARDIoGRAMplusC4D Consortium et al.^14^
rs11591147∗	1p32.3	*PSCK9*	R46L	G/T	1.39	Benn et al.^18^
rs17228212	15q22	*SMAD3*	Intergenic	C/T	1.01	CARDIoGRAMplusC4D Consortium et al.^14^

The *APOA5* promoter variant has been shown to be functional.^19^ All SNPs were included in the 19-SNP gene score (GRS19).

OR, odds ratio; SNP, single nucleotide polymorphism.

∗ SNPs that were selected for the 14-SNP-GRS (GRS14) construction.

**Table 2:** Characteristics of the 537 Afro-Caribbean participants

	Cases (n = 178)	Controls (n = 359)	*P* value
Age, y	63.20 (± 10.50)	51.66 (± 13.54)	< 1 × 10^−5^
Male sex, %	64.0	43.7	< 1 × 10^−5^
Hypertension, %	78.7	30.1	< 1 × 10^−5^
Hypercholesterolemia, %	52.8	15.0	< 1 × 10^−5^
Diabetes, %	53.9	14.8	< 1 × 10^−5^
Current smokers, %	27.5	13.4	< 1 × 10^−5^
BMI	27.41 (± 4.86)	27.15 (± 5.62)	0.535
Obesity (BMI > 30 kg/m^2^), %	23.9	28.9	0.224

Data were available for all participants and are presented as mean (± SD) or percent. Categorical variables were compared using the χ^2^ test. The student *t* test was used for comparison between continuous variables.

BMI, body mass index.

**Table 3:** Risk allele frequencies of the 19 SNPs and GRSs in Afro-Caribbean controls compared with white controls

SNP	Gene	Afro-Caribbeans without CHD (n = 359)	Whites without CHD (n = 1214)	*P* value
RAF	RAF
rs4341	*ACE*	0.6	0.52	8 × 10^−3^
rs662799	*APOA5*	0.13	0.06	1 × 10^−5^
rs1042031	*APOB*	0.15	0.18	0.19
rs429358	*APOE*	0.23	0.17	0.01
rs7412	*APOE*	0.93	0.91	0.23
rs10757274	*CDKN2A/CDKN2B*	0.21	0.48	8.19 × 10^−20^
rs599839	*CELSR2/PSRC1/SORT1*	0.25	0.78	3 × 10^−77^
rs708272	*CETP*	0.76	0.56	1 × 10^−11^
rs1746048	*C × CL12*	0.53	0.86	1 × 10^−40^
rs7025486	*DAB2IP*	0.32	0.26	0.03
rs10455872	*LPA*	0.01	0.07	1 × 10^−5^
rs3798220	*LPA*	0.01	0.02	0.21
rs1801177	*LPL*	0.001	0.01	NA
rs328	*LPL*	0.94	0.90	0.02
rs17465637	*MIA3*	0.24	0.71	2 × 10^−57^
rs9818870	*MRAS*	0.08	0.16	1 × 10^−4^
rs1799983	*NOS3*	0.12	0.33	8 × 10^−15^
rs11591147	*PSCK9*	1.00	0.99	NA
rs17228212	*SMAD3*	0.12	0.31	9 × 10^−13^
Nonweighted GRS19 (SD)		13.17 (± 2.10)	16.59 (± 2.20)	< 1 × 10^−100^
Nonweighted GRS14 (SD)		9.69 (± 1.70)	12.76 (± 1.69)	< 1 × 10^−100^

For white participants, RAFs are reported from the NPHSII cohort.

CHD, coronary heart disease; GRS, genetic risk score; NA, not available; NPHSII, Second Northwick Park Heart Study; RAF, risk allele frequency; SD, standard deviation; SNP, single nucleotide polymorphism.

**Table 4:** Association between cardiovascular risk factors and GRS with CHD in Afro-Caribbeans

Multivariate logistic regression for CHD			
	OR	95% CI	*P* value
Age	1.06	(1.03-1.08)	< 1 × 10^−5^
Male sex	3.09	(1.84-5.19)	2 × 10^−5^
Hypertension	4.30	(2.49-7.26)	< 1 × 10^−5^
Diabetes	2.13	(1.23-3.68)	7 × 10^−3^
Hypercholesterolemia	3.45	(2.08-5.73)	< 1 × 10^−5^
Smoking	3.06	(1.64-5.71)	5 × 10^−4^
Nonweighted GRS19	1.40	(1.09-1.80)	8 × 10^−3^

ORs are expressed per 1 standard deviation change.

CHD, coronary heart disease; CI, confidence interval; GRS, genetic risk score; OR, odds ratio.

**Table 5:** Predictive value of GRSs in Afro-Caribbeans after adjustment with TCRFs

Multivariate logistic regression for CHD (N = 537)			
	OR	95% CI	*P* value
NW_GRS19	1.40	(1.09-1.80)	8 × 10^−3^
W_GRS19	1.20	(0.94-1.53)	0.14
NW_GRS14	1.43	(1.12-1.83)	4 × 10^−3^
W_GRS14	1.25	(0.99-1.59)	0.06

ORs are expressed per 1 standard deviation change and were calculated from model incorporating each GRS, age, sex, hypertension, diabetes, hypercholesterolemia, and smoking.

CI, confidence interval; GRS, genetic risk score; NW, nonweighted; OR, odds ratio; TCRFs, traditional cardiovascular risk factors; W, weighted.

**Table 6:** Predictive values of GRSs in Afro-Caribbeans and whites males after adjustment with TCRFs

	Afro-Caribbeans			Whites (NPHSII)		
OR	95% CI	*P* value	OR	95% CI	*P* value
NW_GRS19	1.62	(1.16-2.27)	4 × 10^−3^	1.27	(1.06-1.51)	9 × 10^−3^
W_GRS19	1.32	(0.96-1.81)	0.08	1.33	(1.11-1.60)	2.3 × 10^−3^
NW_GRS14	1.42	(1.03-1.95)	0.03	1.40	(1.19-1.66)	5.3 × 10^−5^
W_GRS14	1.38	(1.01-1.88)	0.04	1.42	(1.20-1.67)	3.3 × 10^−5^

ORs are expressed per 1 standard deviation change and were calculated from model incorporating each GRS, age, hypertension, diabetes, hypercholesterolemia, and smoking in both ethnic groups. In the Afro-Caribbean sample, we considered only men (n = 271) because the NPHSII cohort included only men (n = 1360 for GRS19 and n = 1607 for GRS14).

CI, confidence interval; GRS, genetic risk score; NPHSII, Second Northwick Park Heart Study; NW, nonweighted; OR, odds ratio; W, weighted.
