# Supplementary material for: Influence of Genetic Risk Factors on Coronary Heart Disease Occurrence in Afro-Caribbeans
Source: Can J Cardiol. 2016 Aug;32(8):978–85. doi: 10.1016/j.cjca.2016.01.004 (PMC4977013; doi:10.1016/j.cjca.2016.01.004)
Supplement: Supplemental Tables S1-S5 and Supplemental Figures S1 and S2 [file mmc1.pdf]

Supplemental table S1: Risk allele frequencies of 19 reported CHD SNP in Afro-Caribbean cases and controls

SNP	Chromosome	Gene	Alleles	Call rate	Risk allele frequency		
			**Risk**/reference	%	**Cases N=178**	**Controls N=359**	**p**
**rs4341**	17q23	*ACE*	**G**/C	95	0.58	0.60	0.66
**rs662799**	11q23	*APOA5*	**G**/A	95	0.14	0.13	0.75
**rs1042031**	2p23-24	*APOB*	**A**/G	95	0.18	0.15	0.37
**rs429358**	19q13.2	*APOE*	**C**/T	96	0.24	0.23	0.79
**rs7412**	19q13.2	*APOE*	**C**/T	97	0.91	0.93	0.41
**rs10757274**	9p21	*CDKN2A/CDKN2B*	**G**/A	94	0.24	0.21	0.43
**rs599839**	1p13.3	*CELSR2/PSRC1/SORT1*	**A**/G	95	0.35	0.25	0.015
**rs708272**	16q21	*CETP*	**C**/T	95	0.74	0.76	0.61
**rs1746048**	10q11.2	*CXCL12*	**C**/T	95	0.63	0.53	0.028
**rs7025486**	9q33	*DAB2IP*	**A**/G	96	0.26	0.32	0.15
**rs10455872**	6q26	*LPA*	**G**/A	94	0.01	0.01	NA
**rs3798220**	6q26	*LPA*	**C**/T	96	0.01	0.01	NA
**rs1801177**	8p22	*LPL*	**A**/G	96	0	0.001	0.88
**rs328**	8p22	*LPL*	**C**/G	97	0.94	0.94	NA
**rs17465637**	1q41	*MIA3*	**C**/A	95	0.31	0.24	0.08
**rs9818870**	3q23.3	*MRAS*	**T**/C	95	0.11	0.08	0.25
**rs1799983**	7q35-36	*NOS3*	**T**/G	95	0.16	0.12	0.2
**rs11591147**	1p32.3	*PSCK9*	**G**/T	94	0.99	1	NA
**rs17228212**	15q22	*SMAD3*	**C**/T	98	0.13	0.12	0.74

Supplemental table S2: Association between GRS and CHD in Afro-Caribbeans.

	Cases N=178	Controls N=359	P
**Non-weighted GRS19**	13.90 (±2.07)	13.17 (±2.10)	$1.6x10^{-4}$
**Weighted GRS19**	1.74 (±0.24)	1.68 (±0.20)	$1.4x10^{-2}$
**Non-weighted GRS14**	10.28 (±1.68)	9.69 (±1.70)	$1.6x10^{-4}$
**Weighted GRS14**	1.66 (±0.23)	1.61 (±0.21)	$9x10^{-3}$

Data are presented as mean (SD)

Supplemental table S3: Association between the 19-SNP GRS and cardiovascular risk factors in Afro-Caribbeans without CHD

**Afro-Caribbean Controls N=359**	**Status** (number)	**Mean weighted GRS**	**P**	**Mean non-weighted GRS**	**P**
**Hypertension**	No (251)	2.54 (0.46)	0.21	13.08 (2.14)	0.32
	Yes (108)	2.61 (0.50)		13.32 (2.06)	
**Diabetes**	No (306)	2.56 (0.47)	0.94	13.18 (2.13)	0.66
	Yes (53)	2.56 (0.51)		13.04 (2.06)	
**Dyslipidaemia**	No (305)	2.56 (0.46)	0.42	13.14 (2.10)	0.80
	Yes (54)	2.61 (0.56)		13.22 (2.24)	
**Smoking**	No (310)	2.57 (0.48)	0.83	13.15 (2.13)	0.92
	Yes (49)	2.55 (0.45)		13.18 (2.09)	

Data are presented as number (SD)

Supplemental table S4: Association between the 19-SNP GRS and cardiovascular risk factors in Afro-Caribbeans with CHD

**Afro-Caribbean cases N=178**	**Status** (number)	**Mean weighted GRS**	**P**	**Mean non-weighted GRS**	**P**
**Hypertension**	No (38)	2.69 (0.52)	0.80	13.87 (2.46)	0.92
	Yes (140)	2.66 (0.48)		13.91 (2.09)	
**Diabetes**	No (82)	2.65 (0.48)	0.66	13.77 (2.25)	0.46
	Yes (96)	2.68 (0.50)		14.01 (2.11)	
**Dyslipidaemia**	No (84)	2.59 (0.49)	0.04	13.40 (2.17)	0.004
	Yes (94)	2.74 (0.48)		14.34 (2.08)	
**Smoking**	No (129)	2.67 (0.50)	0.79	13.94 (2.10)	0.70
	Yes (49)	2.65 (0.45)		13.80 (2.35)	

Data are presented as number (SD)

Supplemental table S5: Risk allele frequencies of the 19 SNPs in the control group of the two study samples and in other populations of European or African ancestry

Gene	SNP	Risk Allele	RAF in Caucasians from NPHS (95%CI)		RAF in Caucasians from HapMAP or 1000 Genomes*	RAF in Afro-Caribbeans (95% CI)		RAF in Afro-Caribbeans from 1000 Genomes	RAF in Africans from HapMap or 1000 Genomes*	RAF in African-Americans from 1000 Genomes
**ACE**	**rs4341**	**G**	0.52	(0.50-0.53)	0.49*	0.60	(0.55-0.65)	0.58	0.59*	0.55
**APOA5**	**rs662799**	**G**	0.06	(0.05-0.07)	0.02	0.13	(0.10-0.17)	0.16	0.13	0.12
**APOB**	**rs1042031**	**A**	0.18	(0.17-0.19)	0.20	0.15	(0.12-0.19)	0.17	0.16	0.20
**APOE**	**rs429358**	**C**	0.17	(0.16-0.18)	0.18*	0.23	(0.19-0.28)	0.25	0.24*	0.20
**APOE**	**rs7412**	**T**	0.09	(0.08-0.10)	0.07	0.07	(0.04-0.10)	0.12	0.09	0.13
**CDKN2A**	**rs10757274**	**G**	0.48	(0.47-0.50)	0.45	0.21	(0.17-0.25)	0.19	0.14	0.29
**PSRC1/*SORT1***	**rs599839**	**A**	0.78	(0.76-0.80)	0.72	0.25	(0.20-0.30)	0.25	0.17	0.20
**CETP**	**rs708272**	**C**	0.56	(0.53-0.59)	0.59*	0.76	(0.72-0.80)	0.75	0.81*	0.75
**CXCL12**	**rs1746048**	**C**	0.86	(0.84-0.88)	0.85	0.53	(0.48-0.58)	0.44	0.47	0.52
**DAB2IP**	**rs7025486**	**A**	0.26	(0.24-0.28)	0.32	0.32	(0.27-0.37)	0.33	0.42	0.27
**LPA**	**rs3798220**	**C**	0.02	(0.01-0.03)	0.03	0.01	(0-0.03)	0.00	0	0.05
**LPA**	**rs10455872**	**G**	0.07	(0.07-0.08)	0.08	0.01	(0-0.03)	0.00	0	0.02
**LPL**	**rs328**	**C**	0.90	(0.09-0.11)	0.88	0.94	(0.91-0.96)	0.91	0.97	0.93
**LPL**	**rs1801177**	**A**	0.01	(0.01-0.02)	0.02*	0.001	(0-0.003)	0.06	0.06*	0.03
**MIA3**	**rs17465637**	**C**	0.71	(0.69-0.72)	0.73	0.24	(0.20-0.28)	0.21	0.15	0.29
**MRAS**	**rs9818870**	**T**	0.16	(0.15-0.17)	0.17	0.08	(0.05-0.11)	0.08	0.05	0.10
**NOS3**	**rs1799983**	**T**	0.33	(0.32-0.35)	0.34	0.12	(0.09-0.15)	0.08	0.07	0.11
**PCSK9**	**rs11591147**	**G**	0.99	(0.99-100)	0.97	1	—	0.00	1	1
**SMAD3**	**rs17228212**	**C**	0.31	(0.30-0.32)	0.30	0.12	(0.09-0.15)	0.08	0.14	0.12

Data from HapMap are from HapMap-YRI (Yoruba in Ibadan, Nigeria) for Africans and HapMAP–CEU (western and northern Europe) for Caucasians
Data from 1000 Genomes (marked with an asterix*) are from British population for Caucasians and Yoruba population in Nigeria for Africans.
For African-Americans, all data are from 1000 genomes (South West USA).
RAF: risk allele frequency

Supplemental figure S1: Distribution of 19-SNP GRS categories in Afro-Caribbean and Caucasian controls

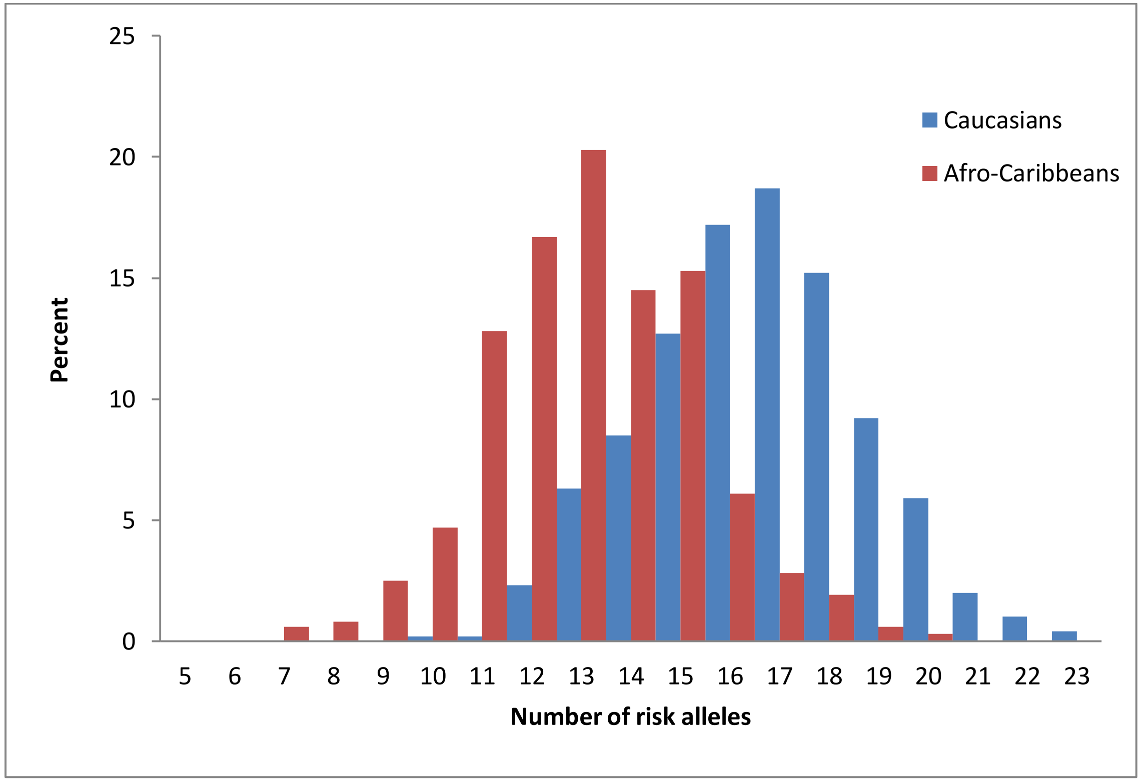

Supplemental figure S2: Incremental contribution of GRS19 to CHD discrimination in Afro-Caribbeans (N=537)

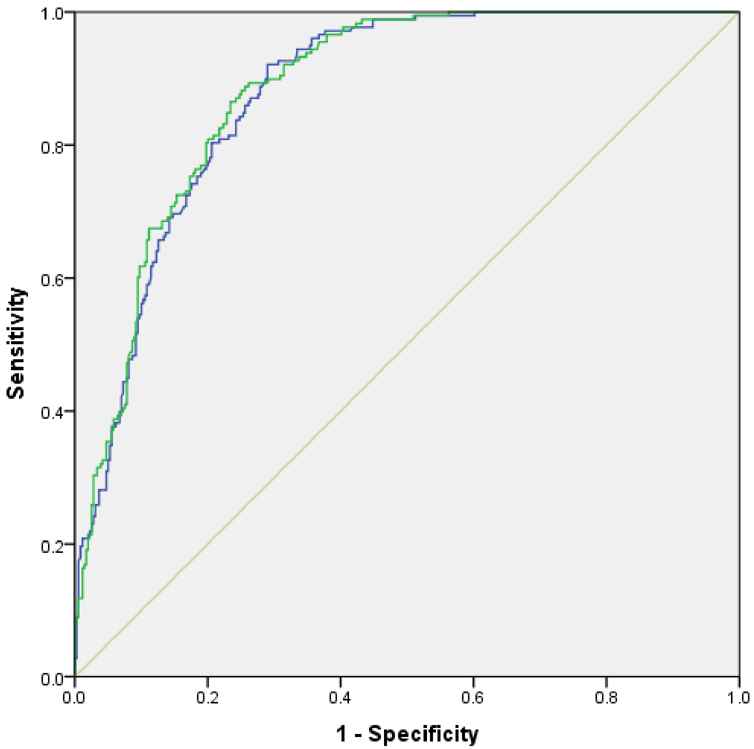

----TCRFs + GRS AUC=0.883 (0.856-0.911)

p=0.19

----TCRFs alone AUC=0.878 (0.850-0.906)

Predictions are based on logistic regression models incorporating TCRFs (age, sex, hypercholesterolemia, diabetes, hypertension, and smoking) alone or with the addition of the non-weighted GRS19.

The area under the curve (AUC) measures the ability of the model to discriminate between subjects with CHD and subjects without CHD
